# Fabrication of Polytetrafluoroethylene Coated Micron Aluminium with Enhanced Oxidation

**DOI:** 10.3390/ma13153384

**Published:** 2020-07-30

**Authors:** Benbo Zhao, Shixiong Sun, Yunjun Luo, Yuan Cheng

**Affiliations:** 1 School of Chemical Engineering and Technology, North University of China, Taiyuan 030051, China; 20170027@nuc.edu.cn (B.Z.); 20190061@nuc.edu.cn (S.S.); 2 School of Materials Science and Engineering, Beijing Institute of Technology, Beijing 100081, China; 3 Key Laboratory for Ministry of Education of High Energy Density Materials, Beijing 100081, China

**Keywords:** PTFE coatings, aluminium, enhanced oxidation, aging-resistant performance

## Abstract

Aluminium (Al) powders of micron size are widely applied to energetic materials as a high energy fuel. However, its energy conversion efficiency is generally low due to low oxidation activity. In this paper, a polytetrafluoroethylene (PTFE) coating layer with both protection and activation action was successfully introduced onto the surface of Al via adsorption and following heat treatment. The preparation conditions were optimized and the thermal activity of this core-shell composite material was studied. The potential enhancement mechanism for Al oxidation was proposed. The results showed that PTFE powders deformed into membrane on the surface of Al after the sintering process. This polymer shell could act as an effective passivation layer protecting internal Al from oxidation during aging. The reduction in metallic Al of Al/PTFE was decreased by 84.7%, more than that in original spherical Al when the aging time is 60 days. Moreover, PTFE could react with Al resulting in a thin AlF_3_ layer, which could promote the destruction of Al_2_O_3_ shell. Thus, PTFE could enhance oxidation activity of micro-Al. The conversion of Al was increased by a factor of 1.8 when heated to 1100 °C. Improved aging-resistant performance and promoted oxidation activity of Al could potentially broaden its application in the field of energetic materials.

## 1. Introduction

Al powders have been widely applied to energetic materials especially for solid propellant as a high energy fuel [1,2]. The specific impulse of propellant could be increased as high as 15%, which is attributed to the addition of Al [1]. However, its combustion efficiency is always a problem in aluminized propellant. During combustion, Al spheres cannot be ignited immediately due to the dense Al_2_O_3_ passivation layer with high boiling point. They often accumulate and agglomerate at the burning surface. Consequently, large agglomerates form, the so called coarse oxidation products in condensed combustion products (CCPs) [1,3,4,5]. Many researchers [3,6,7] have focused on the CCPs of propellant. They have found that it determines the values of two phase losses, which can reduce examined *I_sp_* as high as 3–5% [5]. Thereby, activation of Al is worth exploring.

Reducing its size down to nano-scale is the main direction for increasing reaction activity of Al. However, nano-Al powders are much more expensive than micro-Al and possess processing, safety and environmental issues [8]. The aging resistant performance is also a problem of nano-Al. For the foregoing reasons, nano-Al are barely used in fielded propellant formulations. Thereby, activation of micro-Al in a variety of ways such as ball milling [9,10,11], pre-stressing micro-Al [8], coating [12,13], and tailoring surface conditions [14] has gained the interest of researchers. Among these works, coating is considered an effective way.

Many materials such as nickel (Ni) [15,16], glycidyl azide polymer (GAP) [17], nitrocellulose (NC) [18], polyvinylidene fluoride (PVDF) [19,20,21] and so on have been used as shell on Al. The results showed that these shell materials could activate the metallic Al to some extent. Among these materials, fluoride shells stand out due to their excellent activation effect on the Al reaction activity. For example, Gromov et al. [22] studied nano-Al powders passivated by fluoropolymer. They found that the fluoropolymer shell could not only maintain its Al content during aging but also increase the conversion efficiency up to 94% heating to 1400 °C. However, polytetrafluoroethylene (PTFE) with fluorine content as high as 75% have seldom been introduced onto the surface of Al due to its insoluble nature. In fact, the Al/PTFE system is known for its remarkable theoretical reaction enthalpy (21 GJ/m^3^, ~9.7 kJ/g) [1,23]. In addition, PTFE has proven to be an effective polymer on activation of Al in different ways. Levitas and Pantoya [24,25] studied the pre-ignition reaction of Al. It was found that hydrogen bonding had an influence on the reactivity of Al/PTFE. Dreizin et al. [10] and Dolgoborodov et al. [26] studied mechanically activated Al/PTFE and stated that oxidation activity of Al could be dramatically increased in Al/PTFE. There are still many other meaningful works [27,28,29] concerning Al/PTFE system. They proved the obvious activation effect of PTFE on Al. We also studied the Al/PTFE system and similar results were achieved [30,31,32]. However, these Al/PTFE composite materials aforementioned are not spheres, which is important to the mechanical performance of energetic materials. Therefore, coating PTFE onto the surface of micro-Al is a meaningful direction worth exploring.

Yang et al. [13] prepared core–shell type nano-Al/PTFE with largely increased reaction activity. However, the preparation process, in situ polymerization, cannot be extended to micro-Al readily. Kim et al. [12] proposed a facile way to prepare micro-Al/PTFE core-shell composite. The results showed that PTFE coatings could largely promote the oxidation activity of 45 μm Al. This is very important for potential application in the energy field. However, the preparation conditions and oxidation mechanism were not investigated in detail. Inspired by Kim [12], we introduced PTFE onto the surface of Al as a coating via sintering process in this paper. At the same time, something new has been brought in. The preparation conditions were optimized and the potential oxidation mechanism was studied. The prepared PTFE shell could not only decrease the reduction in metallic Al content during aging but also facilitate Al oxidation when heated. The conversion efficiency of micro-Al increased by 1.8 times in a relatively low temperature range. This significantly enhanced oxidation activity of Al could potentially improve the combustion performance of energetic materials.

## 2. Experimental

### 2.1. Materials

Al powders (22 μm) were obtained from Angang Group Aluminium Powder Co., Ltd (Anshan, China). 3M Dyneon TF 5050Z PTFE emulsion (200 nm) was purchased from Minnesota Mining and Manufacturing Co. (St. Paul, MN, USA).

### 2.2. Al/PTFE Composites Preparation

**Figure 1:**
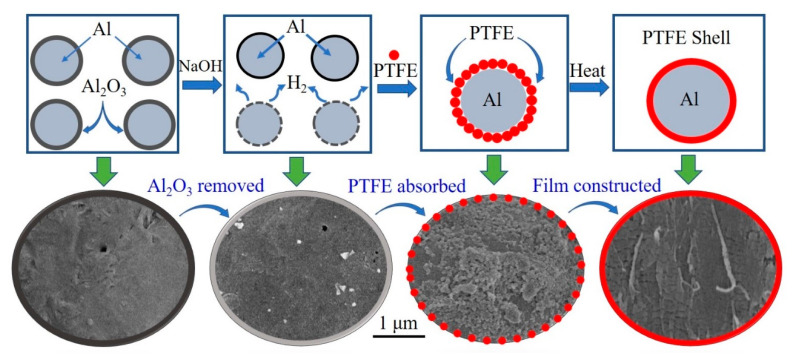
The diagram of the preparation process of aluminium/polytetrafluoroethylene (Al/PTFE) core-shell material. The red spots refer to PTFE particles. The bottom half of the diagram crop from scanning electron microscopy (SEM) images of prepared Al/PTFE.

The passivation layer of Al powders was corroded via 1g/L NaOH solution with magnetic stir. Then the pH value of this aqueous solution was adjusted to 9~10. After that, PTFE emulsion was added to this aqueous with stirring at 600 rpm. The PTFE nano particles could be adsorbed onto the surface of Al due to the Van der Waals force between Al and surfactants. After 30 min adsorption of PTFE particles, the solution stood for 5 min followed by filtration. The obtained product was dried for 12 h in vacuum at room temperature. Then, it was heated step by step in a SK-G08123K tube furnace (Tianjin Central Electric Furnace Co., Ltd., Tianjin, China) in argon (Ar) atmosphere. The curve of programed temperature is shown in Figure S1 (in supplementary materials). The total heating time (t_h_) is 4 h. The obtained product was ground in mortar followed by sieving with a 300 mesh griddle. Then the final PTFE coated Al powders were obtained. A diagram of the preparation process is shown in Figure 1.

### 2.3. Characterization

#### 2.3.1. FTIR

Fourier transform infrared (FTIR) spectra of Al/PTFE were recorded with a Nicolet 8700 FTIR spectrometer (Thermo Fisher Scientific Co., Waltham, MA, USA). Before determination, the prepared samples were ground with potassium bromide (KBr) and pressed at 40 psi pressure. Then the spectra were recorded using a computerized recorder. The FTIR spectra of pure PTFE in emulsion was also determined as a control. Before determination, PTFE was washed with deionized water and dried in vacuum at 80 °C.

#### 2.3.2. SEM Analysis

The morphology characteristics of Al/PTFE was achieved with a scanning electron microscopy (SEM) (Hitachi, S4800, Tokyo, Japan). Prior to analyzation, conductive coating was deposited on the surface of the powders.

#### 2.3.3. Thermal Performance

The reaction performances of PTFE coated Al was studied with thermal gravimetric analyzer (METTLER TOLEDO, TGA STARe System, Zurich, Switzerland). The heating rate is 10 K/min. The determination was conducted in simulated air conditions (N_2_/O_2_ 79/21 volume%), N_2_ and O_2_. The flow rate is 40 mL·min^−1^.

#### 2.3.4. XRD Pattern

To study the oxidation processes of Al, oxidation products recovered at selected temperatures were analyzed by a X-ray diffractometer (XRD) (PANalytical, Eindhoven, Netherland). The recycle operation is similar to Dreizin’s work.

#### 2.3.5. Aging Resistant Test

To determine the aging resistant performance of Al/PTFE powders, accelerated aging was conducted in a GDSJ-100 constant temperature and humidity equipment (Beijing hengtai garfunkel test equipment Co., LTD, Beijing, China). The conditions were: 80 °C, relative humidity 70%.

## 3. Results and Discussion

### 3.1. FTIR

**Figure 2:**
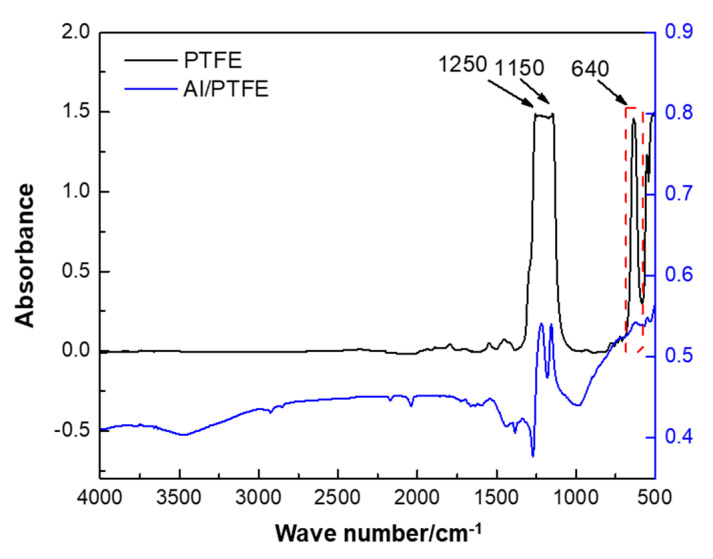
Fourier transform infrared spectroscopy (FTIR) spectra of PTFE coated Al. The PTFE was pressed into a transparent sheet. The Al/PTFE sample was mixed with potassium bromide (KBr) and pressed.

The FTIR spectra of PTFE in the emulsion and PTFE-coated Al is shown in Figure 2. Three characteristic peaks at 1250, 1150 and 640 cm^−1^ [33] can be observed in the FTIR graph of PTFE. The peaks at 1250 and 1150 cm^−1^ could be attributed to the absorption of CF_2_ asymmetrical stretching and CF_2_ symmetrical stretching, respectively. The peak at 640 cm^−1^ could be attributed to the absorption of CF_2_ wagging. These peaks appeared in the spectra of Al/PTFE composite powders as well, which signify that PTFE have been combined with Al. However, from this observation, it was not enough to determine whether PTFE is coated onto the surface of Al. Thereby, some more characterizations on the morphology of Al/PTFE composites were conducted as follows.

### 3.2. SEM Graphs of Al/PTFE Composites.

**Figure 3:**
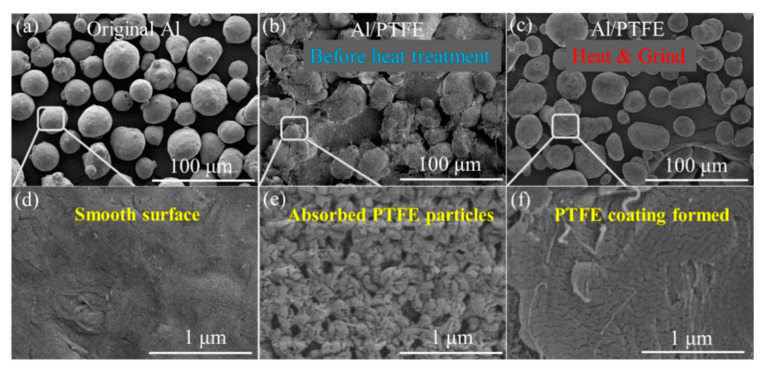
SEM images of Al/PTFE composites: (**a**,**d**) original Al powders; (**b**,**e**) Al/PTFE before heat treatment; (**c**,**f**) Al/PTFE after heat treatment. The maximum heating temperature (T_h_) is 335 °C, the total heating time (t_h_) is 4 h.

The SEM graphs of original Al and Al/PTFE composite materials before & after heat treatment are shown in Figure 3.

**Figure 4:**
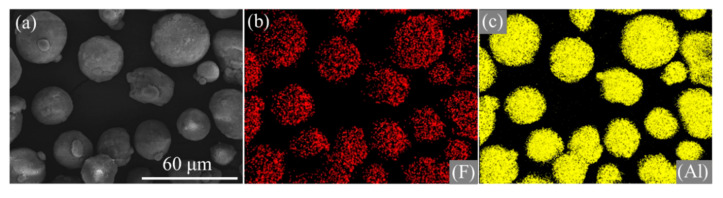
SEM images and elemental maps of Al/PTFE core-shell materials. (**a**) SEM image, (**b**) F, (**c**) Al. The preparation conditions of Al/PTFE were T_h_ = 335 °C, t_h_ = 4 h.

It is obvious that original Al powders are spheres of 20–30 um with a smooth surface. After being stirred in aqueous solution, a large amount of PTFE particles was adsorbed onto the surface of Al. There might be two main kinds of interaction forces contributing to this adsorption. One is the electrostatic interaction between Al and F atom. The other is the Van der Waals force between Al and surfactants on the surface of PTFE particles. Based on the fact that the polarity of PTFE molecule is very low due to the symmetrical structure, the Van der Waals force between Al and surfactants dominates the interaction force. The particles are of hundreds of nanometers in accordance with the size from the supplier (200 nm). After heat treatment, these nano particles melted and deformed to film as shown in Figure 3c,f. Al/PTFE powders before heated were contacted and could hardly flow when the container was tilted. Those PTFE particles are sensitive to shear forces. They would be large flakes if they were ground at this time. After the sintering process, no obvious changes occurred in their external morphology. However, these joined Al/PTFE powders became a little bit hard and brittle due to the deformation and crystallization of PTFE during heat treatment. After they were ground and sieved by a 300 mesh griddle, most agglomerates were separated to independent spheres. Then Al/PTFE core-shell materials with evenly PTFE coatings were achieved. Some more SEM graphs of Al/PTFE composites are shown in Figure S2. Energy dispersive spectrometer (EDS) analysis was also conducted to prove the film on Al surface is PTFE as shown in Figure 4.

Figure 4 indicates that F signals dispersed similarly to that of Al. In addition, the signals of F and Al coincide with the distribution of Al/PTFE composites. Based on the results of SEM, EDS and FTIR analysis, it could be concluded that nano PTFE particles in emulsion were adsorbed onto the surface of Al and deformed to film during heat treatment.

### 3.3. Process Optimization of Al/PTFE Composites

**Figure 5:**
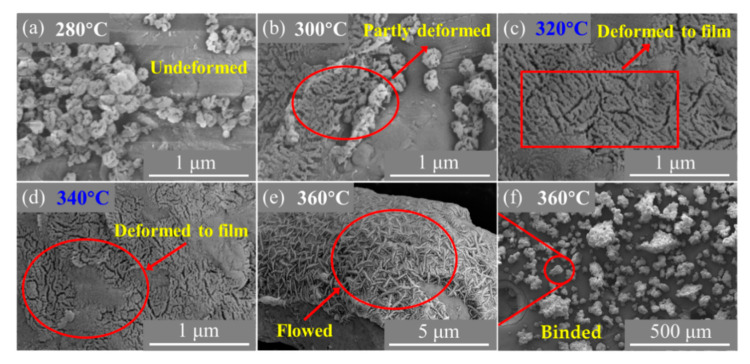
SEM images of Al/PTFE composites heated at different temperatures: (**a**) 280 °C; (**b**) 300 °C; (**c**) 320 °C; (**d**) 340 °C; (**e**,**f**) 360 °C. t_h_ = 4 h.

According to the results of morphology characterization, it could be inferred that the temperature of heat treatment is of vital importance for the morphology of the Al/PTFE core-shell material. Thereby, the optimal process temperature was investigated via varying it from 280–360 °C by increasement of 20 °C. The SEM images of prepared Al/PTFE are shown in Figure 5.

Figure 5 shows that PTFE nano particles cannot change their shape when the maximum temperature of treatment is 280 °C. When it increased to 300 °C, some of PTFE particles deformed while the others retained their shape. Most of those particles melted and joined when they reached 320 °C, which is similar to the morphology characteristic of Al/PTFE at 340 °C. Some accumulated PTFE particles coalesced forming an obvious cap on Al if they were treated at a relatively high temperature (360 °C). There are similarities between the phenomena mentioned above and Kitamura et al.’s work [34]. The accumulated PTFE particles on the Al surface could act as nodes. Below 310 °C the PTFE particles hardly change their shape due to the Van der Waals force and the accumulation microstructure remained. The adhesion of the neighboring particles in a node takes place at 320 °C near the melting point [34]. In addition, at 330 °C the original shape of the particles is almost completely lost forming threadlike structures. During heat treatment, a fibril is formed from a number of these threadlike structures bunched together and these fibers tends to coalesce, forming a membrane [34]. When heated up to 360 °C, PTFE particles could completely melt. However, the fluidity of PTFE molten is relatively low due to the super high molecular weight [35,36]. Thereby, not all the Al/PTFE agglomerations combined together. Overall, the preferred temperature is between 320–340 °C. After some more determination on its morphology, we consider 335 °C to be the best process temperature. This is a little higher than the determined melting point of PTFE (~330 °C) in the TGA/DSC analysis of the PTFE as shown in Figure S3. While the heat treatment temperature reported by Kim et al. [12] is 300 °C. Obviously, it is a little lower than the best temperature.

**Figure 6:**
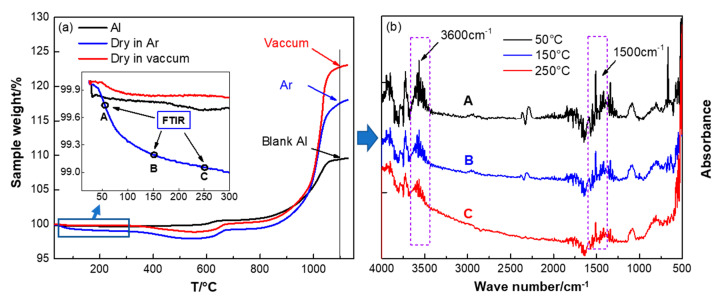
Thermogravimetric analysis (TGA) curves of PTFE coated Al (**a**) and the FTIR spectra of the components in the early stage mass loss (**b**). The testing conditions were, air atmosphere, 10 K/min. The preparation conditions of Al/PTFE were T_h_ = 335 °C, t_h_ = 4 h.

In addition, we found the drying condition could also influence the oxidation of Al/PTFE core-shell materials. The same batch Al/PTFE powders were separated to two parts. One was dried at 80 °C in Ar and the other in vacuum at 20 °C. Figure 6 shows the TGA curves of the Al/PTFE materials in air atmosphere.

Figure 6 shows that the thermal behaviors are different when they are dried at different conditions. When dried at 80 °C in Ar, it is likely to result in mass loss below 300 °C. For the same sample dried in vacuum, the TGA curve is similar to that of original Al powders at low temperature. Thereby, some components may have been included into Al/PTFE samples, and they had not been removed during drying in Ar. To determine the potential components, TGA-IR analysis was conducted. The FTIR spectra at 50, 150 and 250 °C for the sample dried in Ar are shown in Figure 6b. They showed characteristic peaks around 1500 and 3600 cm^−1^, which might be attributed to absorption of the water steam. In addition, the absorption intensity decreased along with increasing of the temperature. It infers that drying in Ar at 80 °C cannot remove all the moisture in Al/PTFE materials readily. This might be caused by the structural hydration on the surface of Al. During preparation of Al/PTFE core-shell structure materials, the Al_2_O_3_ shell was removed by NaOH. H_2_O molecules could be adsorbed at the Al-PTFE interfaces. It is well known that metallic Al with high reaction activity could react with Al resulting in Al(OH)_3_. In addition, this reaction could be facilitated when dried in Ar due to the relatively high temperature (80 °C). Therefore, structural hydration might occur at the surface of Al during preparation of Al/PTFE. This bound water cannot break away from Al readily and stepwise dehydration reaction could occur as illustrated elsewhere [37]. When dried in a vacuum, little structural hydration could occur due to the lower temperature and higher volatilization rate of H_2_O. Thereby, drying in vacuum is preferred for its high efficiency. This is a difference between our work and that of Kim et al. [12].

In addition, from Figure 6 we can see that the mass gains of Al/PTFE were largely promoted by the PTFE compared to that of original Al. That is to say PTFE coatings could enhance the reactivity of Al obviously at low temperature. However, moisture decreased the mass gain of Al/PTFE sample dried in Ar. Two factors may contribute to this reduction in reaction activity. On one hand, the products that resulted from structural hydration would finally form an Al_2_O_3_ layer on the Al surface. This Al_2_O_3_ layer could act as passivation shell and influence the oxidation process of Al. The details could be found in the discussion of thermal properties of Al/PTFE composites (Section 3.4). On the other hand, it was found that chemisorbed H_2_O could stabilize the surfaces of Al_2_O_3_ polymorphs [38]. That is to say the transition between Al_2_O_3_ polymorphs, which is of vital importance in the Al oxidation process, might be suppressed. Thus, the chemisorbed H_2_O may also influence the Al oxidation process. Overall, vacuum drying is preferred.

### 3.4. Thermal Properties of Al/PTFE Composites

**Figure 7:**
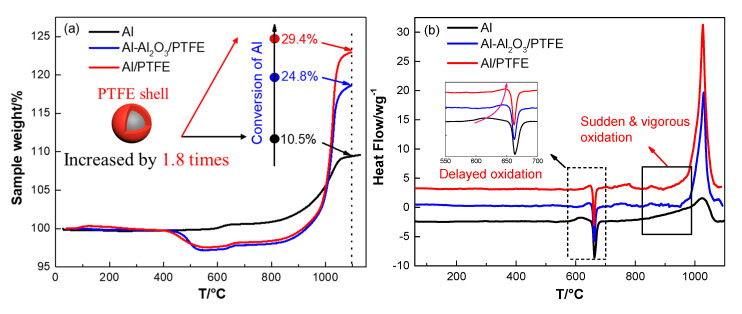
TGA/DSC curves of pure Al, Al-Al_2_O_3_/PTFE and Al/PTFE in air atmosphere, (**a**) TGA; (**b**) Differential scanning calorimetry (DSC). The testing conditions were air atmosphere, 10 K/min.

To determine the reaction activity of PTFE coated Al, thermal analysis was conducted in air atmosphere at elevated temperatures. It is worth noting that Al-Al_2_O_3_/PTFE sample in which the Al_2_O_3_ shell on Al powders was not removed was also prepared and analyzed as a control. Figure 7 shows that Al powders oxidize in three distinct stages in 30–1100 °C similar to Dreizin’s work [39].

The first stage is in 30–550 °C during which the thickness of natural amorphous alumina layer on Al surface increases [40]. No obvious heat flow was observed in this stage due to the slow oxidation. At about 550 °C, amorphous alumina layer transformed into γ-Al_2_O_3_, which could not cover all of the aluminum surface. Thus, the oxidation of Al is obvious in stage II. Then the oxidation rate decreases when the Al_2_O_3_ shell becomes continuous [39]. A little exothermic peak can be found in this stage as shown in Figure 7b. The third stage refers to the temperature range 650–1100 °C. In this stage, the oxidation rate continuously increases. This stage can be divided roughly into two parts: 1) below 960 °C γ-Al_2_O_3_ partially transformed into θ-Al_2_O_3_ and δ-Al_2_O_3_. Little Al was oxidized, resulting in about 4% mass increase along with little heat release; 2) different polymorphs of Al_2_O_3_ transformed into stable α-Al_2_O_3_. Driven by the force resulted from shell shrinkage and expansion of inner Al [40], molten Al could penetrate outside through the flaws. Then, it was oxidized immediately. This stage ended when a dense oxide shell formed and oxidation rate reduced abruptly. The oxidation process for Al-Al_2_O_3_/PTFE in the first stage was the same with that of pure Al. In the second stage, the oxidation for Al-Al_2_O_3_/PTFE was delayed due to the carbon residue produced during PTFE pyrolysis. The exothermic peak was also delayed from 620 °C to 650 °C. In the third stage, the oxidation of Al was largely promoted by the PTFE shell. The conversion of Al was increased from 10.5% to 24.8% when temperature elevated to 1100 °C. However, the amount of reacted Al in Al-Al_2_O_3_/PTFE is similar to pure Al below 960 °C. This phenomenon may be due to the inhibition of AlF_3_ layer that resulted from the fluorination of Al [16]. Then the molten Al was oxidized obviously when it was contacted with air at about 1020 °C. As for Al/PTFE, the oxidation process is similar to Al-Al_2_O_3_/PTFE. However, the mass increase and the output of heat are more obvious. It may be caused by the influence of Al_2_O_3_ layer thickness. It was thinner for Al/PTFE powders when they were heated to about 960 °C since the original Al_2_O_3_ shell was removed. When transforming into α-Al_2_O_3_, the thinner Al_2_O_3_ layer could provide more pores or cracks. In addition, the molten Al could overflow more readily. Thus, the reaction activity of Al/PTFE powders are a little higher than that of Al-Al_2_O_3_/PTFE sample. The conversion ratio of Al (heated to 1100 °C) in Al/PTFE powders increased by 1.8 times from 10.5% to 29.8% when compared with that of pure Al. This result demonstrates that coating of Al with PTFE layer is an effective way of promoting reaction activity of micro-Al.

**Figure 8:**
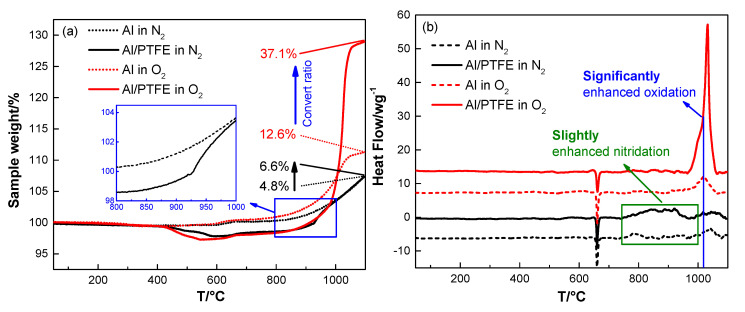
TGA/DSC curves of Al and Al/PTFE in N_2_ and O_2_ atmosphere, (**a**) TGA; (**b**) DSC. The heating rate is 10 K/min.

In addition, N_2_ may also participate in the reaction of Al when it is heated in air. It is not taken into account in the discussion of the reaction process due to the low reactivity [41]. To confirm the correctness of this idea, both Al/PTFE and pure Al were heated in N_2_ and O_2_. The TGA/DSC curves and corresponding parameters are shown in Figure 8 and Table S1, respectively. From Figure 8 we can see that the conversion ratio of pure Al increased by 1.6 times from 4.8% (in N_2_) to 12.6% (in O_2_). That is to say oxidation dominates the reaction in air for pure Al. It may be caused by the difference in diffusion coefficient [42]. O_2_ molecules are smaller, and the polarity of O atoms are closer to Al_2_O_3_ shell. These two factors make O_2_ diffuse into Al_2_O_3_ shell more readily. Thus, the oxidation of Al dominates the reaction. Moreover, the heat release of Al oxidation is higher than that of nitration. This may also contribute to the higher activity of Al oxidation. It is worth noting that the AlN could be transformed to Al_2_O_3_ [41]. Therefore, oxide is the main reaction product. For Al/PTFE powders, PTFE layer slightly increased the conversion ratio of Al in N_2_ from 4.8% to 6.6%, while it was increased by 1.9 times from 12.6% to 37.1% in O_2_. This may be mainly caused by the activation effect from AlF_3_ evaporation. The molten Al oxidizes vigorously when it overflows through α-Al_2_O_3_ shell at about 1020 °C. At this time, the temperature is close to the sublimation point of AlF_3_ (1277 °C) [1]. The vigorous oxidation of Al promotes AlF_3_ sublimation. As a possibility, the AlF_3_ may vanish at this temperature. It will promote Al oxidation largely in return. Consequently, the conversion ratio of Al in Al/PTFE powders is dramatically increased. Overall, the activity of Al nitration is relatively low and oxidation dominates the reaction in air.

### 3.5. Oxidation Mechanism of Al/PTFE Composites

**Figure 9:**
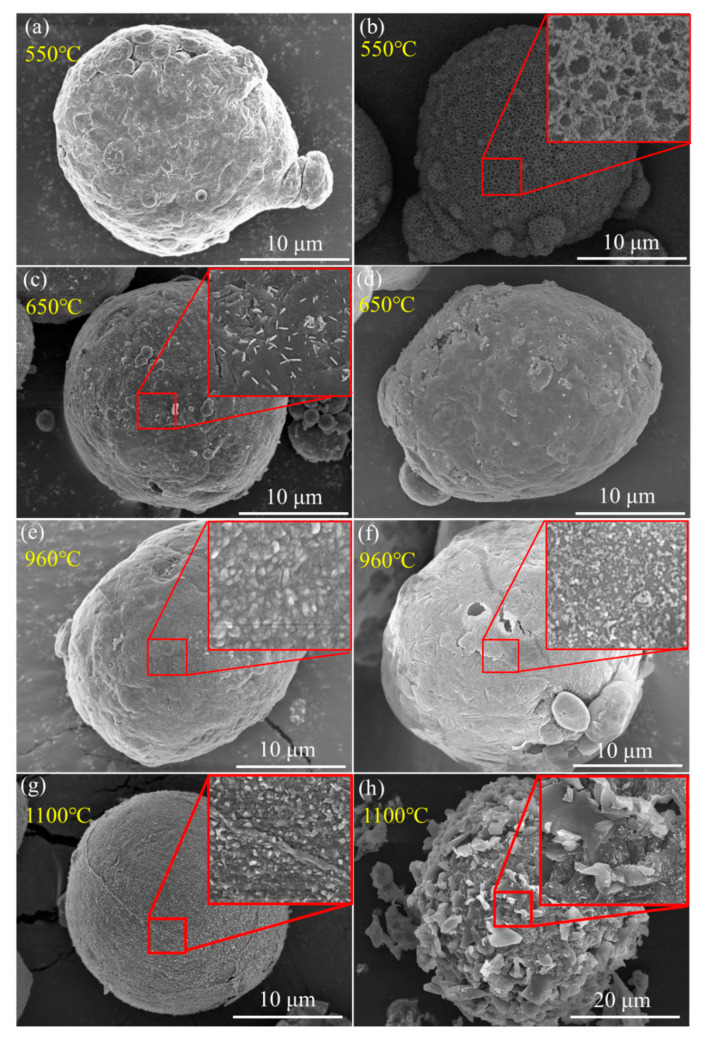
SEM graphs of recovered Al (**a**,**c**,**e**,**g**) and Al/PTFE (**b**,**d**,**f**,**h**) powders at different temperatures. (**a**,**b**) 550 °C; (**c**,**d**) 650 °C; (**e**,**f**) 960 °C; (**g**,**h**) 1100 °C. The samples were recovered from TGA analysis.

**Figure 10:**
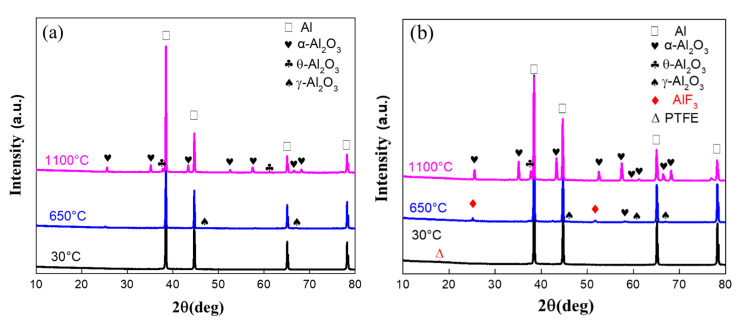
XRD patterns of recovered Al and Al/PTFE powders. (**a**) recovered Al; (**b**) recovered Al/PTFE

**Figure 11:**
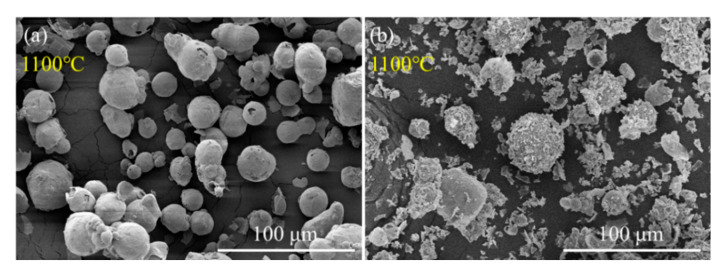
SEM graphs of recovered Al (**a**) and Al/PTFE (**b**) powders at 1100 °C. The samples were recovered from TGA analysis.

**Figure 12:**
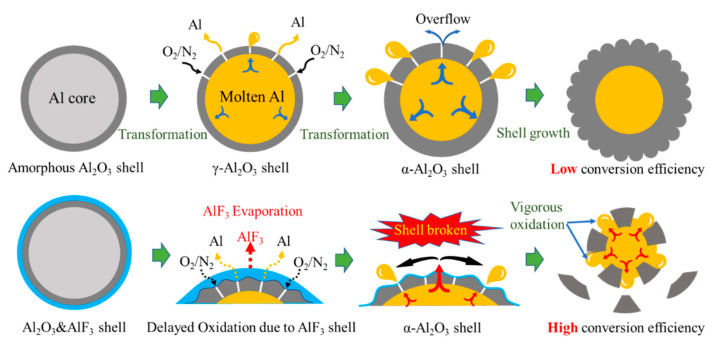
The diagram of potential oxidation promotion mechanism. The dark gray parts refer to Al_2_O_3_. The yellow parts refer to molten Al. The light blue parts refer to aluminum fluoride.

Kim et al. [12] proposed a potential oxidation promotion mechanism at 2016. However, there is not much practical evidence to support their idea. In order to study the reaction process of Al/PTFE further, partially oxidized Al was recovered and analyzed. The SEM images of recovered samples are shown in Figure 9. At about 550 °C, pure Al began to oxidize, while the PTFE layer had not decomposed completely. That is why the oxidation in Al/PTFE powders was delayed in thermoanalysis. At about 650 °C, PTFE on Al surface disappeared. In addition, suspected diffraction peaks of aluminum fluoride appeared as shown in Figure 10. It infers that PTFE reacted with Al. This is because the CF_x_ and COF_x_ radicals resulting from PTFE are very active [24,43,44], and the fluorination of Al could occur readily accompanied by exothermic processes. At about 960 °C, molten Al overflowed through the voids and flaws forming particle oxide products on the surface [40,45]. It is worth noting that the particle oxide products for Al/PTFE are smaller than that of Al. In addition, it is consistent with the DSC result that the heat release of Al/PTFE is not as obvious as that of pure Al at 800–960 °C. This may be due to the AlF_3_ phase, which may hinder the overflow of molten Al. At about 1100 °C, stick oxide products covered the Al surface. This is due to the voids and cracks on the surface of Al resulting from shell shrinkage and expansion of molten Al. As for Al/PTFE, the oxide shell was completely broken as shown in Figure 11. That is to say molten Al overcame the restriction of Al_2_O_3_. Then, it could oxidize vigorously in accordance with the TGA/DSC results. Diffraction peaks of aluminum fluoride disappeared at this temperature. Based on the SEM images, TGA/DSC results and XRD pattern, a potential oxidation promotion mechanism could be raised as shown in Figure 12. For micro-Al, oxidation occurs mainly due to the voids, drawbacks and cracks resulting from the transformation of different polymorphs of Al_2_O_3_ [39]. For Al/PTFE, the early stage oxidation mainly depends on the same reasons. However, the Al_2_O_3_ shell may be broken completely at 900–1100 °C under the influence of shell shrinkage due to Al_2_O_3_ transformation [40,45], expansion of molten aluminum [45], and AlF_3_ sublimation. Molten Al could be immediately oxidized when contacting with air. Consequently, the conversion ratio of Al could be largely increased and the heat release could be dramatically promoted. Overall, Al powders could be activated, caused by the PTFE layer, which is of significant importance to its application in energetic materials.

### 3.6. Aging Resistant Performance of Al/PTFE

Aging resistant property is a key performance for Al powders in energetic formulations. Thus, artificial accelerated aging of Al was carried out at 60 °C for 60 days with humidity level of 70%. The active Al content as shown in Figure S5 was determined by oxidation-reduction titration. The pure Al was blocked after just 10 days of aging. In addition, the active Al content was decreased 18.9% from 99.2% to 80.5%. This is related to the polarity of the Al_2_O_3_ shell. H_2_O and O_2_ molecules can form ions on the Al_2_O_3_ shell surface due to the strong polarity. Driven by the electric field of Al shell, charged ions could diffuse through lattice defects forming an ion current. Then inner Al could be oxidized along with the ion current diffusing inward through the oxide layer. Therefore, the active Al content declined obviously along with aging. As for Al/PTFE, the PTFE layer could form a polymer insulation due to its low polarity nature. It could interdict the ion current effectively. Thus, the active Al content in Al/PTFE was decreased only 2.9%, which is 84.7% lower than that for pure Al after aging 60 days. This is a further study and development for Al/PTFE material compared to that of Kim et al. [12].

At present, micro-Al powders are applied to energetic materials such as propellants, pyrotechnics and explosives. It can be used up to 18% in composite solid propellant. However, the low combustion efficiency limits its broader application. In Al/PTFE core-shell structured material, the PTFE layer could largely increase the oxidation activity of Al. The agglomeration of Al could be decreased during combustion. Based on this advantage, the combustion performance of aluminized propellant could be improved. The energy output efficiency of aluminized explosive could also be increased. Higher percentage application of Al in energetic materials becomes possible in future. Moreover, the PTFE polymer layer could promote the aging-resistant performance. It makes the application of Al possible in some special energetic formulas in which Al cannot be used due to the reaction between Al and H_2_O.

## 4. Conclusions

In this work, PTFE shell layer was coated onto the surface of micro-Al powders. The reaction characteristics were studied based on which potential oxidation mechanism was proposed. Nano PTFE particles could be adsorbed onto the surface of Al with Al_2_O_3_ shell removed in an aqueous solution. During sintering, the PTFE particles could form a thin layer on the surface of Al. This polymer shell acts as an effective passivation layer protecting internal Al from oxidation during aging. The reduction in metallic Al of Al/PTFE was decreased by 84.7% more than that in the original spherical Al when they are aged for 60 days. In addition, the decomposition products of PTFE are very active. They react with Al resulting in a thin AlF_3_ layer, which can concentrate the oxidation of Al below 1100 °C. Then, the Al_2_O_3_ shell can be completely broken under the influence of shell shrinkage due to Al_2_O_3_ transformation, expansion of molten aluminum and AlF_3_ evaporation. Molten Al could be immediately oxidized when it is contacted with air. Consequently, the conversion ratio of Al could be largely increased and the heat release could be dramatically promoted. That is to say the reaction activity of micro-Al is largely increased. The conversion of Al was increased by a factor of 1.8 when it was heated to 1100 °C compared to control. Overall, this polymer shell acts as an effective passivation layer as well as an activation agent for oxidation. Improved aging-resistant performance and promoted oxidation activity of Al may potentially broaden its application in energetic materials.

## Supplementary Materials

The following are available online at https://www.mdpi.com/1996-1944/13/15/3384/s1, Figure S1: The curve of programed temperature in the heat treatment of Al/PTFE, Figure S2: SEM images of core-shell structure Al/PTFE composite materials, Figure S3: TGA (a) and DSC (b) curve of PTFE nano particles in aqueous solution, Figure S4: The active Al content in pure Al and Al/PTFE powders during aging, Table S1: Parameters of TGA/DSC curves of Al and Al/PTFE.
