# Supplementary material for: Fabrication of Polytetrafluoroethylene Coated Micron Aluminium with Enhanced Oxidation
Source: Materials (Basel). 2020 Jul 30;13(15):3384. doi: 10.3390/ma13153384 (PMC7435380; doi:10.3390/ma13153384)
Supplement: Supplementary file 1 [file materials-13-03384-s001.pdf]

materials

MDPI

Article

Fabrication of Polytetrafluoroethylene Coated Micron Aluminium with Enhanced Oxidation

Benbo Zhao [1], Shixiong Sun [1], Yunjun Luo [2,3,*] and Yuan Cheng [1,*]

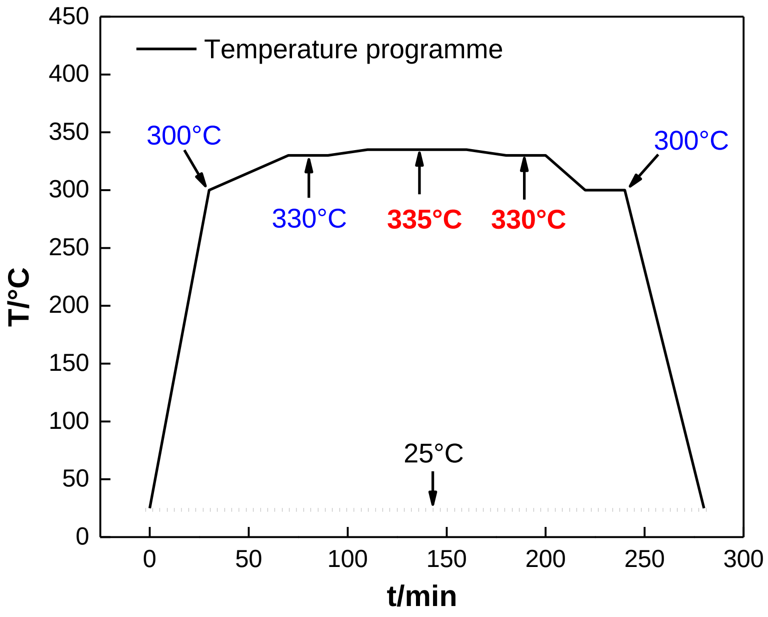

Figure S1. The curve of programed temperature in the heat treatment of Al/PTFE.

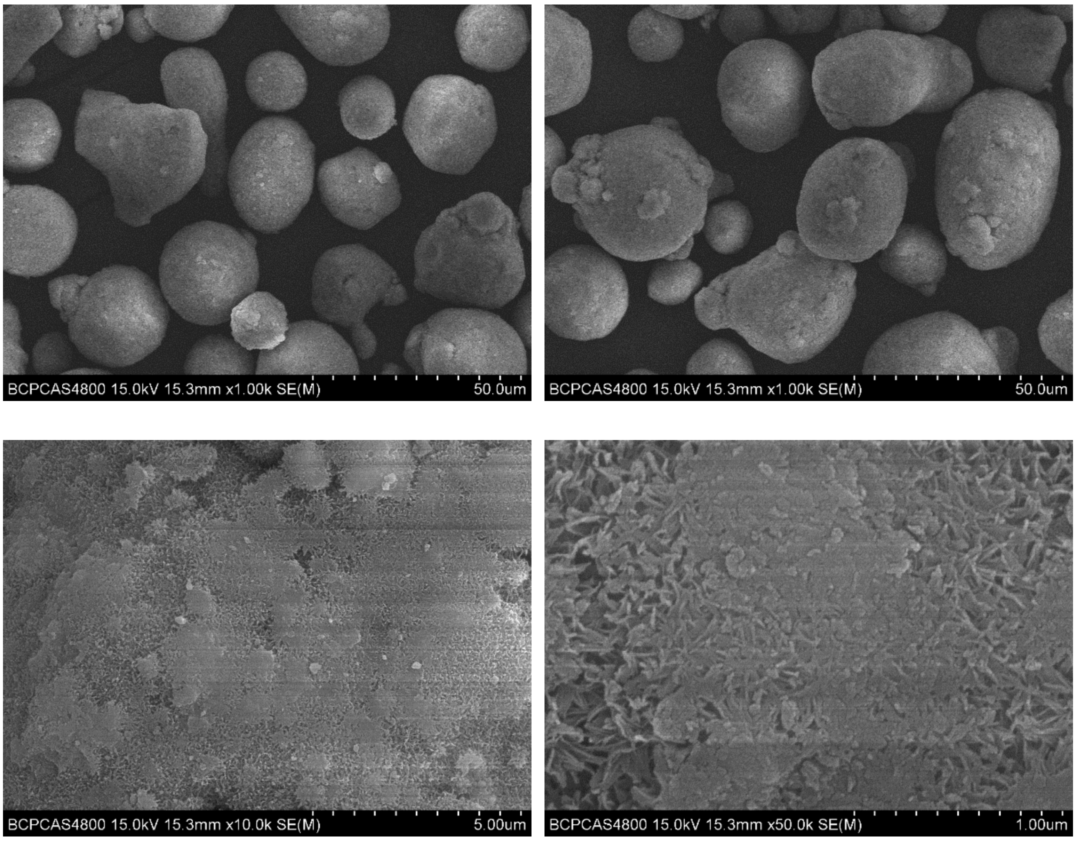

Figure S2. SEM images of core-shell structure Al/PTFE composite materials.

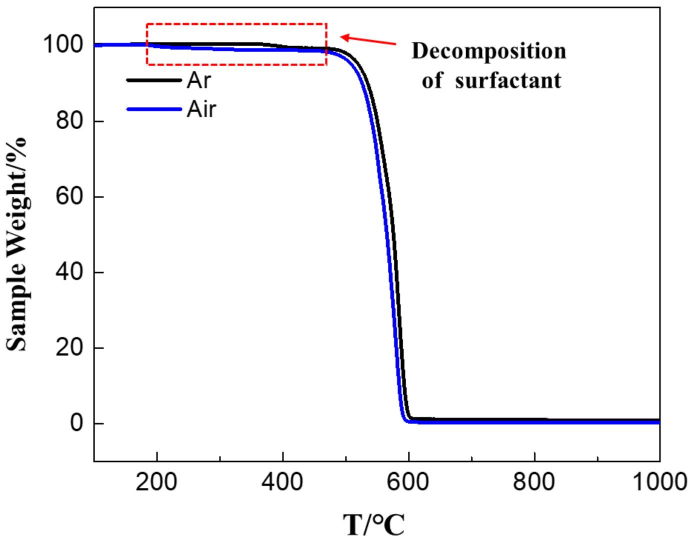

(a) TGA

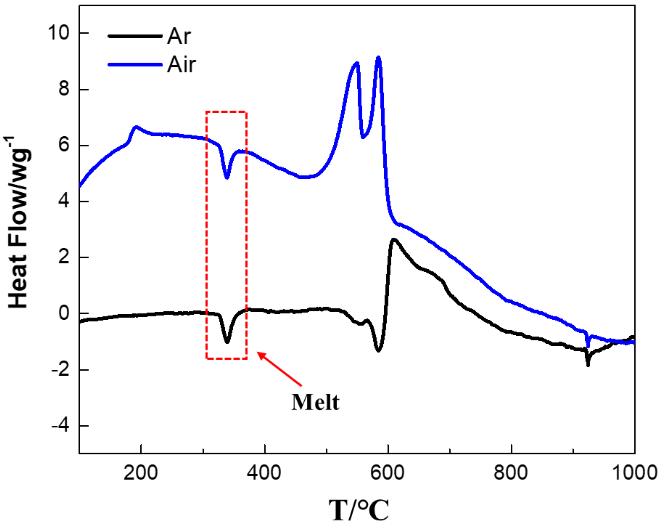

(b) DSC

Figure S3. TGA (**a**) and DSC (**b**) curve of PTFE nano particles in aqueous solution.

Table S1. Parameters of TGA/DSC curves of Al and Al/PTFE.

Sample	Atmosphere	Convert Ratio/%	Reaction Heat/(kJ·g^{-1})
Al	N_2	4.83	2.19
Al	O_2	12.6	3.04
Al	Air	10.5	2.43
Al/PTFE	N_2	6.61	4.39
Al/PTFE	O_2	37.1	10.4
Al/PTFE	Air	29.4	8.02

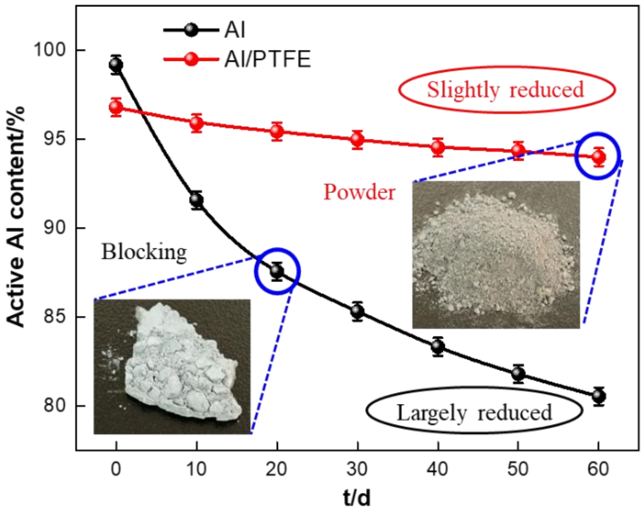

Figure S4. The active Al content in pure Al and Al/PTFE powders during aging.
